# Student Self-Assessments in Clinical Operative Dentistry: Bridging Perception and Performance

**DOI:** 10.1055/s-0045-1809914

**Published:** 2025-07-09

**Authors:** Danya Hashem, Abeer Farag, Rania Zahran Mubarak, Amnah A. Algarni, Nisreen Nabiel Hassan, Anfal Alqussier, Somaya Ali Saleh

**Affiliations:** 1 Department of Restorative Dental Science, College of Dentistry, Taibah University, Madinah, Saudi Arabia; 2 Department of Restorative Dentistry, Faculty of Dentistry, Minia University, Minya, Egypt; 3 Department of Operative Dentistry, Faculty of Dentistry, Ain Shams University, Cairo, Egypt

**Keywords:** self-assessment, faculty assessment, operative dentistry, cavity preparation

## Abstract

**Objectives:**

Student self-assessment in operative dentistry plays a crucial role in the development of clinical skills and professional competence. This study aimed to investigate the agreement between students' self-assessment and faculty assessment in clinical operative dentistry and correlate this with the student’s academic achievement and year of study.

**Materials and Methods:**

Data were collected from 126 female undergraduate 4th and 5th year dental students who conducted self-assessment of class II cavity preparations following minimal invasive principles and composite resin restorations on patients followed by evaluation by two calibrated and independent evaluators using a standardized rubric. Numerical data were presented as mean and standard deviation values. Agreement was analyzed using intra-class correlation coefficient and correlations were analyzed using Spearman's rank order correlation coefficient.

**Results:**

For 4th-year students, there was no significant difference between students' self-assessment and faculty scores regarding retention form and shade matching ( *p* > 0.05), while for other parameters students' self-assessment scores were significantly higher than faculty scores ( *p* < 0.05). For 5th-year students, self-assessment scores were significantly higher within all parameters ( *p* < 0.05). There was no significant correlation between academic achievement (written exam scores) and the self-assessment scores in the clinical exam ( *p* > 0.05) of both academic years.

**Conclusion:**

Students from both 4th and 5th years of study tend to overestimate the assessment of their cavity preparations and restorations; however, there is no correlation between this and their level of academic achievement.

## Introduction

There are various methods to evaluate student performance. The conventional approach involves faculty members giving either formative or summative feedback to students, which is the primary method employed in dental schools. Alternatives like peer assessment and self-assessment are less frequently utilized, even though they are encouraged by educators and academic organizations. 1 2 Self-assessment fosters shared educational responsibility and encourages reflective learning among students. It is particularly beneficial in formative learning, as it directs students' efforts toward areas of knowledge or skills they have yet to master. Additionally, it enables students to gain a realistic understanding of their strengths and weaknesses, guiding them toward responsible self-directed learning. 1 3 Therefore, self-assessment by students is fundamental to developing the skills necessary for achieving the best outcomes in patient-centered care. 4 This is especially important in operative dentistry where, in the initial stages of developing hand skills, students need to comprehend how to attain the expected outcomes according to established grading criteria, typically centered around an ideal preparation. 5 6 Indeed, operative dentistry is a discipline abundant in essential objective criteria for evaluation. Tooth preparations possess distinct attributes, such as depth and angles, that can be easily measured. Therefore, it serves as an excellent area for examining students' self-assessment skills and how these relate to their technical performance, abilities, and knowledge. 7 Students' self-assessment is not a new concept in critical evaluation, and numerous research studies have concentrated on enhancing student learning outcomes through self-assessment, both in quantitative and qualitative terms. 8 However, these research studies provide inadequate information about any kind of structured training of students on how to perform a self-assessment. Also, there is limited information on the effect of continuum of training students to self-assess across the entire curriculum. 8

The gradual development from beginner to skilled dental care practitioners should involve students' self-assessment scores becoming closer to those of faculty assessments by the end of their professional training. It is important that the assessment scores of students and faculty not only align but also show some degree of correlation by the conclusion of the students' training. 4

The Commission on Dental Accreditation (CODA) states that “Graduates must demonstrate the ability to self-assess, including the development of professional competencies and the demonstration of professional values and capacities associated with self-directed, lifelong learning.” 9 With this perspective, self-assessment has been incorporated as part of the clinical operative dentistry curriculum at Taibah University College of Dentistry throughout the year. Therefore, this retrospective study aims to investigate the agreement between the students' self-assessment and the faculty assessment and correlate this with the student's academic achievement (written exam score) and year of study. This is important to evaluate the level of accuracy of self-assessment as it is a fundamental feature for practicing dentists and is a significant skill to enhance in dental students. Furthermore, this research can give an insight on whether the skill of accurate self-assessment is demonstrated in Taibah University's dental students. The null hypothesis is that there is no difference in the level of agreement between the student self-assessment and the faculty evaluators' assessment of a class II cavity preparations and composite restorations conducted during the operative dentistry clinical final exam at Taibah University College of Dentistry. Furthermore, there is no correlation between the student academic year of study and academic achievement with the level of accuracy of self-assessment.

## Materials and Methods

### Study Design, Participants, and Setting

This study was approved by Taibah University, College of Dentistry Research Ethics Committee with reference number (TUCDREC/ 22042021/DHashem). Sample size calculation was performed based on the results of a previous study 10 and the minimum total required sample was found to be 103 students. Data were collected from 126 female undergraduate dental students; 61 juniors enrolled in year 4 and 65 seniors in year 5 Clinical Operative courses at the Taibah University College of Dentistry during the academic years 2020 to 2023. In the clinical operative course, self-assessment is part of the curriculum where students assess their cavity preparations and restorations prior to being evaluated by the supervising faculty member for each clinical procedure throughout the year. A rubric with pre-determined assessment criteria for each type of tooth preparation and restoration is routinely used in the clinical operative courses each year. This rubric is explained at the start of each academic year to students and all faculty members participating in the course and they are trained and calibrated on using the rubric before the start of the operative clinical sessions. The 4th year dental students are trained and calibrated in a preclinical laboratory for 1 month and undertake a competency exam on a plastic tooth before starting the clinical sessions. For standardization, data used in this study were student self-assessments of class II cavity preparations of upper or lower posterior teeth on patients who were diagnosed with normal pulps or reversible pulpitis with moderate to deep carious lesions detected using X-rays. This was conducted during a timed final exam at the end of the year for students in year 4 and year 5. After cavity preparation was completed using minimal invasive technique, students performed self-assessment by conventional visual observation using a mirror and explorer and recorded their scores using the criteria in the rubric. Two calibrated faculty evaluators specializing in Operative Dentistry then assessed the cavity preparation using the same standardized rubric. After completing the composite resin restoration (Tetric N-Ceram, Ivoclar Vivadent, Liechtenstein), students again performed self-assessment followed by the two faculty evaluators. The faculty evaluators performed the assessment independently and were blinded to the self-assessment score given by the student whereas they recorded their evaluation on a separate paper. The average score of the two faculty was taken. The two calibrated faculty evaluators were the same throughout the study time. The rubrics used for cavity preparation and composite resin restoration assessment are displayed in Tables 1 and 2 , respectively.

**Table 1:** Clinical assessment rubric for cavity preparation

Cavity preparation			
Items	Correct item (2 marks for each item)	Correctable with slight modification (1 mark for each item)	Uncorrectable error (0 mark for each item)
**Outline form**	- Extended to include all the lesion and conservative - Rounded sweeping curves Conservatively includes pits & fissures if necessary	- Under extended outline - Sharp line angles that could be rounded without over-extending the outline - Remaining active soft carious lesion at margin	- Over-extended outline - Very sharp line angles that could not be corrected without over-extending the outline
**Resistance form**	- Proper cavo-surface angle (CSA) inclination - No undermined or friable enamel margins - No undermined cusp(s) Bulk resistance - Smooth and flat walls and floors - No sharp line angles	- Sharp line angles that could be rounded without weakening the tooth structure - Undermined or friable enamel margins that could be removed without weakening the tooth structure - CSA less or more than 90 that could be corrected without weakening the tooth structure - Shallow depth	- Over-extended outline - Very sharp line angle - Extremely undermined wall(s) - Extremely undermined weakened cusp
**Retention form**	- Proper CSA inclination/bevel - Each part of compound or complex cavity has its own independent retention	−	- Non-retentive cavity - Over-divergent wall of the preparation
**Caries management & pulpal floor**	- Complete removal of all infected soft carious dentin - Proper cavity for deep lesions, removal of deep carious lesion in spot removal without deepening the pulpal floor - Preserving sufficient viable dentin bridge	- Incomplete removal of carious lesion - Shallow pulpal floor - Irregular, rough, and tilted floors - Nonselective removal of deep lesion (increasing the cavity depth)	- Unnecessary over-extended cavity depth - Violating the healthy sound dentin bridge - Iatrogenic pulp exposure
**Finishing of walls**	- Smooth, flat with proper CSA inclination - No undermined enamel except for labial walls of class III preparations - Smoothly rounded line angles	- Sharp line angles that could be rounded without weakening the tooth structure - Undermined or friable enamel margins that could be removed without weakening the tooth structure - CSA less than 90 that could be corrected without weakening the tooth	- Sharp line angles that could not corrected without destruction of sound tooth structure - Extremely rough wall(s) - Extremely undermined wall(s)

**Table 2:** Clinical assessment rubric for composite resin restoration

Composite resin restoration			
Item	Achieved expected outcome (2 marks for each item)	Doesn't meet expected outcome (1 mark for each item)	Uncorrectable error (0 mark for each item)
**Color matching**	Match the shade and translucency of adjacent tooth tissues	Does not match the shade and translucency of adjacent tooth tissues, but the mismatch is within the normal range of tooth shades	Does not match the shade and translucency of adjacent tooth tissues, but the mismatch is outside the normal range of tooth shades
**Anatomy, contact & contour**	Anatomy replicates the functional contralateral tooth Properly located proximal contact, buccal or lingual contours	Over-featured anatomy Over-tight or shifted proximal contact Over-bulged buccal or lingual contours	Flat anatomy Open proximal contact or/and flat buccal and lingual contours
**Margins**	Flashed with tooth structure	Marginal flashes or overhangs	Marginal ditches
**Occlusion**	Proper	Under-carving with premature contacts	Over-carved and out of occlusion
**Surface finish**	Surface texture similar to polished enamel as determined by means of a sharp explorer	Surface texture gritty or similar to a surface subjects to a white stone or similar to a composite containing supra-micron-sized particles	Surface pitting is sufficiently coarse to inhibit the continuous movement of an explorer across the surface

### Statistical Analysis

A power analysis was conducted to ensure sufficient power for performing a two-sided statistical test of the null hypothesis, which states that there is no difference in the level of agreement between students' self-assessments and the assessments made by faculty evaluators of class II cavity preparations and composite resin restorations and no difference between the student's academic year of study and level of agreement.

By adopting an α (α) level of 0.05, a β (β) level of 0.2 (i.e., power = 80%), a null hypothesis value for intra-class correlation coefficient (ICC) of 0.6, and an alternative hypothesis value of ICC of 0.393 based on the results of a previous study, ^10^ the minimum total required sample size ( *n* ) was found to be 103 students. Sample size calculation was performed using R statistical analysis software version 4.3.2 for Windows. 11

Numerical data were displayed as mean and standard deviation values and Shapiro-Wilk's test was used to analyze for normality. The data were found to be non-parametric and were compared using signed rank test. Agreement was analyzed using ICC and its values were interpreted according to Koo and Lee. 12 Correlations between the student's academic year of study and academic achievement (written exam) with the level of accuracy of the self-assessment were analyzed using Spearman's rank order correlation coefficient and their values were interpreted according to Cohen, 2013. 13 Statistical analysis was performed with R statistical analysis software version 4.3.2 for Windows. ^11^

## Results

The study was conducted on 126 students (i.e., 61 4th-year students and 65 5th-year students), who were assessed and given scores by two faculty members. There was an excellent agreement between both evaluators in both academic years (i.e., ICC = 0.995 [0.994:0.996] for 4th year and 0.996 [0.995:0.996] for 5th year), and for each student.

Results of the agreement between students' self-assessment and faculty assessment are presented in Table 3 . Results showed that for 4th-year students, there was no significant difference between students' self-assessment and faculty evaluation scores regarding retention form and shade matching ( *p* > 0.05), while for other parameters students' self-assessment scores were significantly higher than faculty-evaluation scores ( *p* < 0.05). For shade matching, there was a significantly good agreement between both scores (i.e., ICC = 0.797, *p* < 0.05), meanwhile the agreement for retention form was not statistically significant (i.e., ICC = 0.312, *p* > 0.05). For the resistance form, margins, and occlusion, the agreement was poor (ICC < 0.5). For other parameters, the agreement was not statistically significant ( *p* > 0.05).

**Table 3:** Agreement between students' self assessment and faculty members' evaluation

Academic year	Parameter	Scores (Mean ± SD)		Test statistic	*p* -value	ICC (95% CI)
		Students	Faculty members			
**4th year**	***Outline form***	1.87 ± 0.34	1.38 ± 0.58	**497.00**	<0.001*	**0.177 (−0.178:0.453)**
	***Resistance form***	1.92 ± 0.28	1.79 ± 0.45	**55.00**	0.037*	0.360 (−0.039:0.610)*
	***Retention form***	1.85 ± 0.36	1.75 ± 0.43	**93.50**	**0.141**	**0.312 (−0.133:0.584)**
	***Caries management & pulpal floor***	1.84 ± 0.37	1.43 ± 0.64	**342.00**	<0.001*	**0.191 (−0.204:0.477)**
	***Finishing of walls***	1.82 ± 0.39	1.28 ± 0.45	**720.00**	<0.001*	**0.007 (** − **0.275:0.274)**
	***Cavity score***	9.31 ± 1.19	7.56 ± 1.31	**1,411.00**	<0.001*	**0.181 (−0.157:0.453)**
	***Shade matching***	1.98 ± 0.13	1.97 ± 0.18	**1.00**	**1**	0.797 (0.663:0.878)*
	***Anatomy, contact and contour***	1.92 ± 0.28	1.54 ± 0.56	**265.00**	<0.001*	**0.193 (−0.189:0.475)**
	***Margins***	1.80 ± 0.40	1.57 ± 0.50	**207.00**	0.003*	0.348 (−0.039:0.598)*
	***Occlusion***	1.92 ± 0.28	1.70 ± 0.46	**112.00**	<0.001*	0.406 (0.041:0.637)*
	***Surface finish***	1.82 ± 0.39	1.34 ± 0.48	**576.00**	<0.001*	**0.084 (−0.243:0.363)**
	***Restoration score***	9.48 ± 0.79	8.07 ± 1.11	**1,246.00**	<0.001*	**0.101 (−0.164:0.349)**
**5th year**	***Outline form***	1.83 ± 0.38	1.37 ± 0.57	**497.00**	<0.001*	**0.247 (−0.128:0.513)**
	***Resistance form***	1.98 ± 0.12	1.69 ± 0.47	**190.00**	<0.001*	**0.129 (−0.242:0.415)**
	***Retention form***	1.95 ± 0.21	1.72 ± 0.45	**144.00**	<0.001*	**0.218 (−0.180:0.497)**
	***Caries management & pulpal floor***	1.69 ± 0.53	1.26 ± 0.64	**511.50**	<0.001*	0.536 (0.109:0.744)*
	***Finishing of walls***	1.85 ± 0.36	1.32 ± 0.56	**545.50**	<0.001*	**0.177 (−0.163:0.445)**
	***Cavity score***	9.26 ± 0.97	7.34 ± 1.36	**1,596.00**	<0.001*	0.210 (−0.163:0.497)*
	***Shade matching***	2.00 ± 0.00	1.89 ± 0.36	**21.00**	0.026*	**0.000 (−0.570:0.373)**
	***Anatomy, contact and contour***	1.75 ± 0.43	1.25 ± 0.59	**649.00**	<0.001*	0.271 (−0.116:0.537)*
	***Margins***	1.83 ± 0.38	1.63 ± 0.55	**228.00**	0.013*	**0.226 (−0.218:0.515)**
	***Occlusion***	1.92 ± 0.27	1.66 ± 0.51	**136.00**	<0.001*	0.414 (0.050:0.641)*
	***Surface finish***	1.78 ± 0.41	1.37 ± 0.52	**435.00**	<0.001*	**0.281 (** − 0.102:0.543)*
	***Restoration score***	9.29 ± 0.80	7.75 ± 1.51	**1,389.00**	<0.001*	**0.160 (−0.159:0.423)**
**Overall**	***Outline form***	1.85 ± 0.36	1.37 ± 0.58	**1,958.00**	<0.001*	0.213 (−0.079:0.433)*
	***Resistance form***	1.95 ± 0.21	1.74 ± 0.46	**435.00**	<0.001*	0.229 (−0.055:0.442)*
	***Retention form***	1.90 ± 0.29	1.74 ± 0.44	**459.00**	<0.001*	0.259 (−0.027:0.469)*
	***Caries management & pulpal floor***	1.76 ± 0.46	1.34 ± 0.65	**1,662.00**	<0.001*	0.416 (0.085:0.618)*
	***Finishing of walls***	1.83 ± 0.37	1.30 ± 0.51	**2,488.00**	<0.001*	**0.104 (−0.126:0.306)**
	***Cavity score***	9.29 ± 1.08	7.44 ± 1.34	**5,952.00**	<0.001*	0.196 (−0.133:0.446)*
	***Shade matching***	1.99 ± 0.09	1.93 ± 0.29	**28.00**	0.015*	0.272 (−0.021:0.483)*
	***Anatomy, contact and contour***	1.83 ± 0.37	1.39 ± 0.59	**1,716.00**	<0.001*	0.286 (−0.033:0.507)*
	***Margins***	1.82 ± 0.39	1.60 ± 0.52	**851.00**	<0.001*	0.286 (0.009:0.489)*
	***Occlusion***	1.92 ± 0.27	1.68 ± 0.48	**480.50**	<0.001*	0.409 (0.133:0.594)*
	***Surface finish***	1.80 ± 0.40	1.36 ± 0.50	**1,982.50**	<0.001*	0.189 (−0.090:0.406)*
	***Restoration score***	9.38 ± 0.80	7.90 ± 1.34	**5,209.00**	<0.001*	0.146 (−0.112:0.361)*

Abbreviation: ICC, intra-class correlation coefficient.

Note: *Significant ( *p* < 0.05).

For 5th-year students, self-assessment scores were significantly higher than faculty scores in all parameters ( *p* < 0.05). For caries management and pulpal floor, there was a significantly moderate agreement between both scores (i.e., ICC = 0.536, *p* < 0.05), while for cavity score, anatomy, contact and contour, occlusion, and surface finish, the agreement was poor (ICC < 0.5, *p* < 0.05). For other parameters, the agreement was not statistically significant (p > 0.05).

For both years collectively, students' self-assessment scores were significantly higher in all parameters ( *p* < 0.05). Except for finishing of the walls, there was a significantly poor agreement between both scores (ICC < 0.5, *p* < 0.05).

Results of the correlations between academic achievement (written exam score) and the self-assessment scores for the students of both years are presented in Table 4 . There was no significant correlation between the written exam and self-assessment scores ( *p* > 0.05).

**Table 4:** Correlations between academic achievement (written exam) and self-assessment scores

Academic year	Variables	Correlation coefficient (95% CI)
**4th year**	***Written exam – restoration score***	**0.133 (−0.123:0.373)**
	***Written exam – cavity score***	**−0.206 (−0.435:0.049)**
**5th year**	***Written exam – restoration score***	**−0.028 (−0.270:0.218)**
	***Written exam – cavity score***	**−0.046 (−0.287:0.200)**
**Overall**	***Written exam – restoration score***	**0.034 (−0.141:0.208)**
	***Written exam – cavity score***	**−0.128 (−0.297:0.048)**

Results of the comparisons of the differences in students' self-assessment and faculty-evaluation scores for the same students in both academic years are presented in Table 5 . Results showed that for anatomy and contact/contour, the difference between student's self-assessment score and faculty score measured in 5th year was significantly higher than 4th year ( *p* = 0.020). However, for other parameters, the difference was not statistically significant ( *p* > 0.05). For restoration score, there was a moderate positive correlation between both years' differences that was statistically significant (rs = 0.457, *p* < 0.05), while for other parameters, the correlations were not statistically significant ( *p* > 0.05).

**Table 5:** Difference and correlation between 4th- and 5th-year self-evaluation

Parameter	Difference from faculty members (Mean ± SD)		Test statistic	*p* -value	Correlation coefficient (95% CI)
	4th year	5th year			
**Outline form**	0.56 ± 0.59	0.49 ± 0.55	**109.00**	**0.552**	**0.087 (−0.227:0.384)**
**Resistance form**	0.15 ± 0.36	0.17 ± 0.38	**30.00**	**0.802**	**−0.004 (−0.312:0.304)**
**Retention form**	0.20 ± 0.40	0.29 ± 0.46	**37.50**	**0.301**	**0.089 (−0.225:0.386)**
**Caries management & pulpal floor**	0.59 ± 0.67	0.44 ± 0.50	**185.00**	**0.276**	**−0.056 (−0.357:0.257)**
**Finishing of walls**	0.73 ± 0.45	0.56 ± 0.59	**192.00**	**0.183**	**−0.165 (−0.450:0.150)**
**Cavity score**	2.00 ± 1.45	1.85 ± 1.30	**245.50**	**0.794**	**0.112 (−0.203:0.405)**
**Shade matching**	0.00 ± 0.00	0.07 ± 0.26	**0.00**	**0.149**	**NA**
**Anatomy, contact and contour**	0.29 ± 0.56	0.61 ± 0.59	**84.00**	0.020*	**−0.069 (−0.369:0.244)**
**Margins**	0.32 ± 0.47	0.27 ± 0.45	**95.00**	**0.655**	**−0.058 (−0.359:0.254)**
**Occlusion**	0.24 ± 0.43	0.27 ± 0.45	**56.00**	**0.821**	**0.041 (−0.270:0.344)**
**Surface finish**	0.59 ± 0.50	0.44 ± 0.50	**93.50**	**0.141**	**0.246 (−0.067:0.515)**
**Restoration score**	1.17 ± 1.02	1.49 ± 1.16	**116.00**	**0.118**	0.457 (0.174:0.670)*

Abbreviation: NA, not applicable.

Note: *Significant ( *p* < 0.05).

## Discussion

Successful self-assessment of clinical competency is essential for dentists, as it allows a clear grasp of skills and the detection of inadequacies. 14 Numerous factors may impact a dental student's ability to self-assess, including academic achievement, personality, gender, cultural background, and the incorporation of dental technologies. These variables are critical in students' capacity to gauge their work and progress. 15

The current study aimed to evaluate the agreement between students' self-assessment and faculty assessment during the final clinical operative exam which included a class II cavity preparation and composite restoration and to associate this with student's academic achievement and year of study. A standardized rubric was used for this study which is the same rubric used in the operative curriculum throughout the year. Accurate self-assessment requires the use of a comprehensive grading rubric that clearly defines criteria aligned with learning objectives and ideal performance standards in both preclinical and clinical settings. A previous review indicated that while most self-assessment studies employed grading rubrics, it was often unclear whether students were provided with explicit criteria. 8 This study utilized the same grading rubric that the students used in the operative clinical curriculum for both academic years indicating first, a continuum of training students to self-assess across the entire curriculum, and second, a mechanism to measure students' ability to improve their assessments from the beginning to the end of the curriculum. 8

The results of this study showed both 4th- and 5th-year students' self-assessment scores were significantly higher within most parameters in the rubric compared with faculty evaluation. This is in line with the results of earlier studies that reported a general trend of overestimation of students' self-assessments even with the use of enhanced technology such as CAD/CAM. 5 14 15 16

For example, a study conducted in Japan found that students tend to overestimate their performance in a prosthodontic exercise giving higher self-assessment scores compared with faculty. 15 The study suggested that this finding may be due to increased self-confidence which could lead students to rate their self-assessments more favorably. 15 Another study evaluated the self-assessment scores of 3rd-year dental students performing various operative procedures in a preclinical setting. The students tend to overestimate their self-assessment for all the procedures as compared with actual scores given by faculty. 16

This finding may suggest that students often assess their performance based on memory and personal confidence, which can lead to overestimation. This overconfidence is not necessarily linked to actual performance but to their subjective sense of achievement. 8 17 18

The results also showed no significant correlation between academic achievement (written exam scores) and self-assessment scores of students of both academic years. However, previous studies reported that students with low performance tend to overestimate themselves, which could be due to unawareness of their weaknesses. In contrast, students with high performance tend to underestimate themselves. Students who often underestimate their abilities frequently exhibit low self-esteem and a restricted attitude toward patients, which may be perceived as a lack of competence. 14 15 16

A potential explanation for the non-significant correlation between academic achievement and self-assessment scores found in this study is that both 4th- and 5th-year students were assessed during their final exam at the end of the year. That was a timed exam on class II cavity preparation and composite resin restoration placement, where the students were mostly stressed. Test-related anxiety may affect self-assessment especially for 4th-year students as the operative clinical course is one of the first clinical courses they practice. Another explanation of students' overestimation could be that they believe that rating themselves first will affect the overall score assigned by their evaluators although the evaluating faculty were blinded to the student self-assessment score. 16

Compared with the 4th-year students, 5th-year students' scores were significantly higher when assessing anatomy, contact and contour of their restorations but not for the other parameters. This finding may be attributed to the fact that 5th-year students have more self-esteem and believe their work is improving as they gain more experience.

Self-assessment ability could be enhanced by utilizing pre-defined standardized evaluation criteria with intensive training of students to appraise their work both preclinically and clinically as conducted in this study. It will be more effective to encourage correct self-assessment as a skill in and of itself. 8 10 14 A complete analysis of each parameter offers students vital feedback, identifying their strengths and areas for improvement. 19 Furthermore, it is critical to have standardized teaching and student performance grading criteria, especially during competency examinations. 16 The two involved faculty evaluators in the current study taught during the operative clinical courses and remained consistent throughout the study even in the final exam. Continuous training with reflective feedback leads to improvement of the student's ability to make objective judgments and encourages the development of self-regulation skills, such as controlling their thoughts, emotions, and behaviors during self-assessments. 4 19

The current study does not demonstrate the effect of gender on self-assessment since the sample includes only female students, which is considered a limitation of the study. However, earlier studies showed that both genders tend to overestimate their performance with males overestimating significantly more in some procedures such as class II amalgam preparation. 15 16 Another limitation of the current study is the inclusion of one class II preparation and composite restoration per student. This was done with the aim of standardization of the clinical procedure. Future research should analyze students' capacity to self-assess in operative dentistry with more variables, such as different cavity preparations, and varied restorative material placement in anterior and posterior teeth. Despite these limitations, the study's findings highlight the need for structured self-assessment training to improve accuracy.

## Conclusion

The first null hypothesis stating that there is no difference in the level of agreement between the student's self-assessment and the faculty evaluators' assessment of class II cavity preparations and composite restorations was rejected. The second null hypothesis stating that there is no correlation between the student's academic year of study and academic achievement with the level of accuracy of the self-assessment was accepted. Students from both 4th and 5th years of study tend to overestimate the assessment of their cavity preparations and restorations; however, there is no correlation between this and their level of academic achievement. When integrated properly into dental education, self-assessment can contribute significantly to the development of skilled, self-aware, and reflective practitioners.

## Clinical Relevance

Accurate student self-assessment is fundamental for the development of clinical skills and professional competence. Adding self-assessment practices in the operative curriculum ensures that graduates are better prepared for effective patient care in their future dental practices.

## Notes

**Note:** The affiliation of Anfal Alqussier has been updated as per the request from the first and corresponding author, Danya Hashem, received on July 12, 2025.

## References

[1] Curtis D A Lind S L Dellinges M Setia G Finzen F C Dental students' self-assessment of preclinical examinations J Dent Educ 2008 72 03 265 277 18316530

[2] ADEA Commission on Change and Innovation in Dental Education Hendricson W D Andrieu S C Chadwick D G Educational strategies associated with development of problem-solving, critical thinking, and self-directed learning J Dent Educ 2006 70 09 925 936 16954414

[3] Alfakhry G Mustafa K Ybrode K Scaffolding self-regulated learning in operative dentistry through self-assessment trainingJ Med Educ Curric Dev2024 1123821205241226820 10.1177/23821205241226820 PMC10807336 38268727

[4] Metz M J Durski M T O'Malley DeGaris M Student self-assessment of operative dentistry experiences: a time-dependent exercise in self-directed learning J Dent Educ 2017 81 05 571 581 28461634 10.21815/JDE.016.020

[5] Mays K A Levine E Dental students' self-assessment of operative preparations using CAD/CAM: a preliminary analysis J Dent Educ 2014 78 12 1673 1680 25480283

[6] Mittal P Jadhav G R Pawar M Impact of self-assessment on dental student's performance in pre-clinical conservative dentistry course BMC Oral Health 2024 24 01 593 38778282 10.1186/s12903-024-04140-w PMC11112931

[7] Lee C Asher S R Chutinan S Gallucci G O Ohyama H The relationship between dental students' assessment ability and preclinical and academic performance in operative dentistry J Dent Educ 2017 81 03 310 317 28250037

[8] Mays K A Branch-Mays G L A systematic review of the use of self-assessment in preclinical and clinical dental education J Dent Educ 2016 80 08 902 913 27480701

[9] Commission on Dental Accreditation Accreditation Standards for Dental Education Programs Chicago American Dental Association 2015

[10] AlRahabi M K AlKady A M Ghabbani H M Agreement between faculty member assessments and student self-assessments in a preclinical endodontic programme Aust Endod J 2019 45 03 346 351 30632229 10.1111/aej.12324

[11] R Core Team. A Language and Environment for Statistical Computing. R Foundation for Statistical Computing, Vienna, 2021

[12] Koo T K Li M Y A guideline of selecting and reporting intraclass correlation coefficients for reliability research J Chiropr Med 2016 15 02 155 163 27330520 10.1016/j.jcm.2016.02.012 PMC4913118

[13] Cohen J Statistical Power Analysis for the Behavioral Sciences2nd ed. New York: Routledge; 2013

[14] Huth K C Baumann M Kollmuss M Hickel R Fischer M R Paschos E Assessment of practical tasks in the Phantom course of Conservative Dentistry by pre-defined criteria: a comparison between self-assessment by students and assessment by instructors Eur J Dent Educ 2017 21 01 37 45 26642844 10.1111/eje.12176

[15] Liang L Nagasawa M Ha V Association between gender and self-assessment skills amongst Japanese dental students J Dent Sci 2024 19 03 1533 1539 39035302 10.1016/j.jds.2023.12.025 PMC11259621

[16] Kornmehl D L Patel E Agrawal R Harris J R Ba A K Ohyama H The effect of gender on student self-assessment skills in operative preclinical dentistry J Dent Educ 2021 85 09 1511 1517 33990132 10.1002/jdd.12638

[17] Iguchi A Hasegawa Y Fujii K Student potential for self-assessment in a clinical dentistry practical training course on communication skills Med Sci Educ 2020 30 04 1503 1513 34457818 10.1007/s40670-020-01061-5 PMC8368263

[18] Blanch-Hartigan D Medical students' self-assessment of performance: results from three meta-analyses Patient Educ Couns 2011 84 01 3 9 20708898 10.1016/j.pec.2010.06.037

[19] Daghrery A Alwadai G S Alamoudi N A Students' performance in clinical class II composite restorations: a case study using analytic rubrics BMC Med Educ 2024 24 01 1252 39497127 10.1186/s12909-024-06261-w PMC11536952

